# MRI patterns and clinical outcomes in cerebral palsy: insights from a large MRICS-based cohort

**DOI:** 10.1186/s11689-025-09661-1

**Published:** 2025-12-30

**Authors:** Junying Yuan, Kejie Cao, Dong Li, Jiefeng Hu, Xuejie Wang, Wending Xin, Lingling Zhang, Yiran Xu, Changlian Zhu

**Affiliations:** 1 Henan Pediatric Clinical Research Center and Henan Key Laboratory of Child Brain Injury, Institute of Neuroscience and Third Affiliated Hospital of Zhengzhou University, Zhengzhou, 450052 China; 2 Cerebral Palsy Rehabilitation Center, Third Affiliated Hospital of Zhengzhou University, Zhengzhou, China; 3 Center for Brain Repair and Rehabilitation, Institute of Neuroscience and Physiology, University of Gothenburg, Gothenburg, 40530 Sweden

**Keywords:** Cerebral palsy, MRI, MRICS, Gross motor function, Intellectual disability, Perinatal adversity, Neurodevelopment

## Abstract

**Background:**

To classify MRI patterns in children with cerebral palsy (CP) using the MRI Classification System (MRICS) and examine their associations with perinatal risk factors and clinical outcomes.

**Methods:**

This retrospective cohort study included 1,403 children with CP who underwent post-neonatal cranial MRI between 2011 and 2020. MRI patterns were categorized using MRICS. We analyzed the associations between MRI findings and perinatal risk factors (e.g., gestational age, birth weight, sex, perinatal adversity, plurality) using univariate and multivariable multinomial logistic regression. Clinical outcomes—including CP subtype, gross motor function, intellectual disability, epilepsy, and composite impairment index—were assessed using chi-square, Kruskal–Wallis tests, and correspondence analysis.

**Results:**

MRI abnormalities were observed in 86.5% of children, with predominant white matter injury (PWMI) being most common (46.5%). Preterm birth and perinatal adversity significantly increased the risk of PWMI and PGMI. PWMI was linked with spastic CP, better motor outcomes, and lower rates of intellectual disability. In contrast, PGMI and maldevelopments were associated with epilepsy, hearing loss, and severe impairment. Importantly, a subset of children with normal MRI findings still exhibited substantial functional impairments, emphasizing the limitations of structural imaging alone.

**Conclusions:**

MRI patterns, as classified by MRICS, provide critical insight into the neurodevelopmental heterogeneity of CP. Normal MRI findings do not preclude significant clinical impairment, underscoring the need for integrated neuroimaging and clinical-genetic assessment in CP management.

## Background

Cerebral palsy (CP) refers to a group of permanent disorders affecting movement and posture, attributed to non-progressive disturbances occurring in the developing fetal or infant brain. It is frequently accompanied by impairments in sensation, cognition, communication, and behavior. As the preferred neuroimaging modality, magnetic resonance imaging (MRI) offers a high diagnostic yield for children with CP, enabling precise identification of structural abnormalities and insights into the timing and nature of the brain injury [1, 2]. Previous studies consistently report that over 80% of children with CP exhibit abnormal MRI findings [3–10].

To standardize interpretation of these findings, the MRI Classification System (MRICS) was developed by the Surveillance of Cerebral Palsy in Europe. MRICS categorizes neuroimaging results into five groups: white matter injury (WMI), gray matter injury, maldevelopments, miscellaneous patterns, and normal findings [11]. The system has demonstrated strong interrater reliability and has been adopted in several population-based studies [12–15].

Despite its strengths, MRICS remains underutilized in large clinical datasets from Asian populations, and its potential to correlate imaging patterns with clinical outcomes—such as motor function, comorbidities, and overall impairment— remains insufficiently characterized in large Asian cohorts, particularly regarding its predictive value for functional outcomes. Furthermore, the clinical significance of a normal MRI pattern in children with CP remains a subject of debate, as some may exhibit severe disability despite the absence of structural lesions on imaging.

In this study, we applied MRICS to a large cohort of 1,403 children with CP from a hospital-based rehabilitation center in China [16]. Our objectives were to: determine the distribution of MRI patterns; evaluate their associations with perinatal risk factors**,** and examine how these patterns relate to motor function, cognitive impairment, and overall disability. This work aims to deepen understanding of CP heterogeneity and highlight the clinical utility of MRICS in guiding prognosis and diagnostic decision-making.

## Methods

### Study design and participants

This retrospective cohort study included 1,403 children diagnosed with CP who underwent rehabilitation at the Child Rehabilitation Center of the Third Affiliated Hospital of Zhengzhou University between January 1, 2011, and December 31, 2020. These participants were selected from a larger previously described cohort of 2,012 children with CP [16]. For inclusion in the current analysis, children were required to have undergone at least one post-neonatal cranial MRI (defined as imaging performed after 28 days of life) [16]. Exclusion criteria included: (1) absence of brain MRI data (246 cases without brain imaging, 347 cases with only cranial CT); (2) imaging performed only in the neonatal period without follow-up scans (16 cases). The final analytic sample of 1,403 children thus comprised those with confirmed CP and valid post-neonatal MRI data suitable for MRICS classification. This explains the reduction in the sample size from 2,012 in the previous study to 1,403 in this study.

### MRI classification

MRI examinations were performed using either 1.5 T or 3.0 T scanners, following a standardized protocol that included axial and coronal T1-weighted, T2-weighted, and FLAIR sequences, along with axial diffusion-weighted imaging and sagittal T1-weighted sequences. Sedation was administered when necessary [16]. MRI findings were classified according to the MRICS, which categorizes brain images into five major groups: (A) maldevelopments, (B) predominant white matter injury (PWMI), (C) predominant gray matter injury (PGMI), (D) miscellaneous findings, and (E) normal imaging [11]. The classification process involved both written reports by pediatric neuroradiologists and direct image review by pediatric neurologists, all trained in the MRICS system. The method has shown high interrater reliability [17]. Each participant’s imaging was reviewed by at least one pediatric neuroradiologist and one pediatric neurologist. In cases where MRI appeared normal or showed discrepancies with clinical presentation, further consultation was undertaken between specialists, and additional or advanced imaging was considered if necessary. If multiple scans were available and showed inconsistent results, the most recent scan was used for classification. In cases with multiple injury types, the dominant pattern was assigned based on its extent and expected clinical relevance, following MRICS guidelines [11].

### Definition of perinatal adversity

Perinatal adversity was defined by the presence of one or more of the following factors: fetal distress, active resuscitation at birth, moderate or severe neonatal encephalopathy, or emergency cesarean delivery.

### Impairment index

Children were categorized into low, moderate, or high impairment groups according to combined assessments of motor function, intellectual disability, vision, hearing, and epilepsy status, using criteria adapted from Horber et al. [18]. Impairment index data were available only for children older than four years with complete follow-up information.

### Assessment of gross motor function

Gross motor function was evaluated using the Gross Motor Function Classification System (GMFCS) [19].

### Clinical variables

Clinical variables—including CP subtypes, intellectual disability, epilepsy, visual and hearing impairment—were defined according to standardized criteria reported in previous work [16].

### Statistical analysis

Descriptive statistics were used to summarize cohort characteristics. Univariate analyses examined associations between MRI categories and perinatal factors (e.g., gestational age, birth weight, sex, plurality, and perinatal adversity) using chi-square tests. Multivariable multinomial logistic regression was conducted to explore associations between perinatal risk factors and MRICS patterns, with the normal MRI pattern as the reference category. Collinearity was evaluated using the Generalized Variance Inflation Factor. Clinical outcomes (CP subtype, GMFCS level, comorbidities, and impairment index) were analyzed using chi-square and Kruskal–Wallis tests. *P*-values were adjusted using the false discovery rate method. Correspondence analysis was employed to visualize multidimensional relationships between MRI patterns and clinical variables. Statistical analyses were conducted using R version 4.4 with packages rcompanion, ca, vcd, rstatix, nnet and FSA. Figures were generated using ggplot2 and ggsci. A two-sided *p*-value < 0.05 was considered statistically significant. All analyses were pre-specified, and no data-driven modeling was performed.

## Results

### Study population characteristics

A total of 1,403 children with CP who underwent post-neonatal cranial MRI were included in the analysis. The mean age at the time of MRI was 24.0 ± 22.7 months, with a median of 18 months (interquartile range: 10–29). Among these, 35.7% (501/1403) underwent MRI after the age of two years.

### MRI pattern distribution

**Fig. 1:**
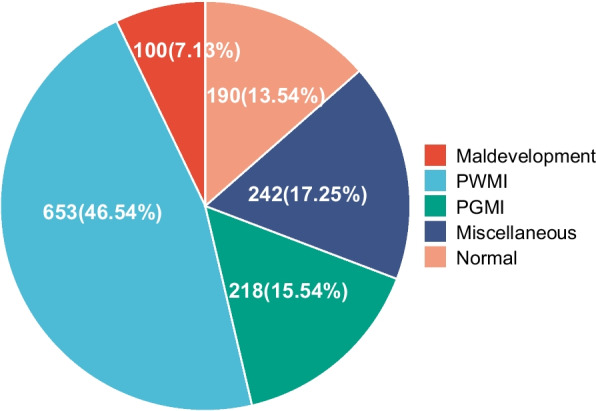
Distribution of MRI patterns. PWMI was the most common MRI pattern, observed in nearly half of the children with CP (46.54%, 653/1403), and followed by miscellaneous pattern and PGMI pattern

MRI abnormalities were identified in 86.5% of the cohort. PWMI was the most frequent pattern, observed in 46.5% of participants. Miscellaneous findings and PGMI accounted for 17.3% and 15.5% respectively. Normal MRI findings were present in 13.5%, while maldevelopments constituted 7.1%. Cortical malformations represented the most common subtype within the maldevelopment group (31%) (Fig. 1).

### Associations with perinatal factors

MRI patterns were significantly associated with gestational age, birth weight, sex, plurality, and perinatal adversity (all *p* < 0.05) (Table 1). Multivariable multinomial logistic regression revealed that, compared to term children with a normal MRI pattern, moderate preterm and very preterm children had 7.38-fold and 9.30-fold increased odds of exhibiting PWMI, respectively. Perinatal adversity was also associated with a higher risk of PWMI (RRR = 1.59, 95% CI: 1.087–2.327). For the PGMI pattern, female sex (RRR = 1.814, 95% CI: 1.178–2.792) and perinatal adversity (RRR = 2.069, 95% CI:1.35–3.172) were significant risk factors. Additionally, moderate preterm birth was associated with a higher likelihood of miscellaneous findings (RRR = 2.199, 1.202–4.023) (Table 2).

**Table 1:** Univariate analysis between MRI results and perinatal factors

	Maldevelopments	PWMI	PGMI	Miscellaneous	Normal	***p***-value
	[%(N/n)]	[%(N/n)]	[%(N/n)]	[%(N/n)]	[%(N/n)]	
Sex						
Male	8.74(39/446)	45.07(201/446)	19.28(86/446)	15.25(68/446)	11.66(52/446)	0.019
Female	6.37(61/957)	47.23(452/957)	13.79(132/957)	18.18(174/957)	14.42(138/957)	
Birth Weight						
≥ 2500 g	8.97(77/858)	30.3(260/858)	21.45(184/858)	21.45(184/858)	17.83(153/858)	< 0.001
< 2500 g & ≥ 1500 g	4.57(18/394)	74.11(292/394)	5.33(21/394)	9.64(38/394)	6.35(25/394)	
< 1500 g	2.48(3/121)	76.03(92/121)	5.79(7/121)	12.4(15/121)	3.31(4/121)	
Gestational Weeks						
≥ 37 wk	9.62(79/821)	27.28(224/821)	22.17(182/821)	21.56(177/821)	19.37(159/821)	
< 37wk & ≥ 32 wk	3.12(10/321)	69.16(222/321)	8.41(27/321)	13.4(43/321)	5.92(19/321)	< 0.001
< 32 wk	3.53(9/255)	80.39(205/255)	2.75(7/255)	8.63(22/255)	4.71(12/255)	
Plurality						
Singleton	7.15(92/1287)	44.29(570/1287)	15.85(204/1287)	18.41(237/1287)	14.3(184/1287)	< 0.001
Multiple	6.14(7/114)	71.93(82/114)	12.28(14/114)	4.39(5/114)	5.26(6/114)	
Perinatal adversity						
No	8.74(72/824)	39.93(329/824)	14.56(120/824)	20.39(168/824)	16.38(135/824)	< 0.001
Yes	4.7(27/575)	56(322/575)	16.87(97/575)	12.87(74/575)	9.57(55/575)

**Table 2:** Multivariate regression of MRICS and perinatal factors

Subtypes	Variables	Coef(SE)	RRR(95% CI)	*p*.value
PWMI	(Intercept)	0.16(0.134)	1.174(0.902, 1.527)	0.233
PWMI	Sex:Female	0.228(0.2)	1.256(0.849, 1.859)	0.254
PWMI	Gestational Age: < 37 & ≥ 32 wk	1.999(0.272)	7.38(4.331, 12.575)	< 0.001^***^
PWMI	Gestational Age: < 32 wk	2.23(0.326)	9.302(4.914, 17.607)	< 0.001^***^
PWMI	Multibirth:Yes	0.475(0.456)	1.608(0.658, 3.928)	0.297
PWMI	Perinatal Adversity:Yes	0.464(0.194)	1.59(1.087, 2.327)	0.017^*^
Maldevelopment	(Intercept)	−0.811(0.183)	0.445(0.311, 0.636)	< 0.001^***^
Maldevelopment	Sex:Female	0.524(0.267)	1.689(1.001, 2.849)	0.05
Maldevelopment	Gestational Age: < 37 & ≥ 32 wk	−0.005(0.428)	0.995(0.43, 2.302)	0.991
Maldevelopment	Gestational Age: < 32 wk	0.269(0.482)	1.308(0.509, 3.364)	0.577
Maldevelopment	Multibirth:Yes	0.717(0.594)	2.048(0.639, 6.562)	0.228
Maldevelopment	Perinatal Adversity:Yes	−0.108(0.284)	0.898(0.515, 1.565)	0.704
PGMI	(Intercept)	−0.308(0.153)	0.735(0.544, 0.993)	0.045^*^
PGMI	Sex:Female	0.595(0.22)	1.814(1.178, 2.792)	0.007^**^
PGMI	Gestational Age: < 37 & ≥ 32 wk	0.053(0.333)	1.054(0.549, 2.024)	0.874
PGMI	Gestational Age: < 32 wk	−1.007(0.502)	0.365(0.137, 0.976)	0.045^*^
PGMI	Multibirth:Yes	0.774(0.519)	2.168(0.784, 6)	0.136
PGMI	Perinatal Adversity:Yes	0.727(0.218)	2.069(1.35, 3.172)	0.0008^***^
Miscellaneous	(Intercept)	0.148(0.14)	1.159(0.881, 1.525)	0.292
Miscellaneous	Sex:Female	0.004(0.224)	1.004(0.648, 1.556)	0.986
Miscellaneous	Gestational Age: < 37 & ≥ 32 wk	0.788(0.308)	2.199(1.202, 4.023)	0.011^*^
Miscellaneous	Gestational Age: < 32 wk	0.585(0.386)	1.795(0.842, 3.825)	0.13
Miscellaneous	Multibirth:Yes	−1.004(0.666)	0.366(0.099, 1.353)	0.132
Miscellaneous	Perinatal Adversity:Yes	−0.055(0.221)	0.946(0.614, 1.459)	0.802

RRR: Relative Risk Ratio ^*^: < 0.5^; **^: < 0.01^; ***^: < 0.001

### MRI patterns and CP subtypes

**Fig. 2:**
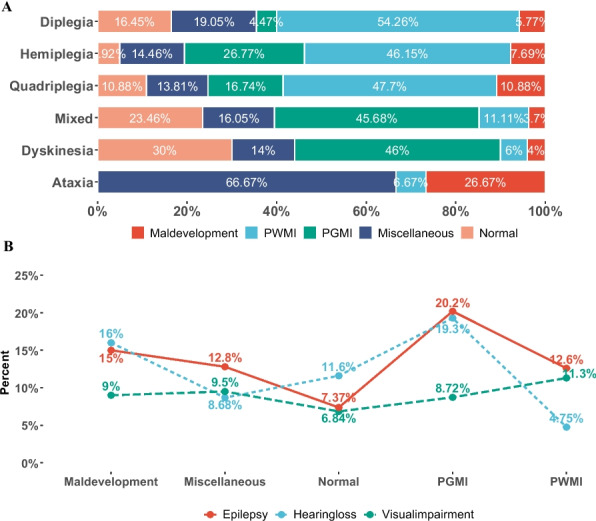
The relationship between MRICS patterns and clinical features: subtypes, epilepsy, hearing loss, and visual impairment. **A** The composite column chart illustrates the distribution of CP subtypes across MRICS patterns. Spastic subtypes—including diplegia, quadriplegia, and hemiplegia—were predominantly associated with the PWMI pattern (54.26%, 47.7%, and 47.7%, respectively). In contrast, mixed and dyskinetic CP subtypes were most frequently associated with the PGMI pattern (45.68% and 46%, respectively). No significant difference was found between mixed and dyskinetic CP in post hoc pairwise comparisons **(**p.adj = 0.845). **B** The line graph shows the prevalence of epilepsy, hearing loss, and visual impairment across MRICS categories. Children with PGMI had the highest rates of epilepsy (20.2%) and hearing loss (19.3%), while PWMI was associated with the lowest rate of hearing loss (4.8%). MRICS patterns were significantly associated with hearing loss and epilepsy (all *p* < 0.05), but no significant association was observed with visual impairment

MRI patterns were strongly associated with CP subtypes, motor function (GMFCS), epilepsy, hearing loss, and impairment index (all *p* < 0.05), while no significant association was observed for visual impairment (*p* = 0.408) (Table 3). PWMI was predominantly linked to spastic subtypes—diplegia (54.3%), hemiplegia (47.7%), and quadriplegia (17.5%). PGMI was more frequently associated with mixed (45.7%) and dyskinetic subtypes (46%) (Table 3, Fig. 2A). There was no statistically significant difference between mixed and dyskinetic types in terms of MRICS distribution (p.adj = 0.845).

**Table 3:** MRICS and clinical features

	Maldevelopments	PWMI		PGMI	Miscellaneous	Normal	* P*
	[%(N/n)]	[%(N/n)]		[%(N/n)]	[%(N/n)]	[%(N/n)]	
Subtypes							
Diplegia	40(40/100)	57.58(376/653)	14.22(31/218)		54.55(132/242)	60(114/190)	< 0.001
Hemiplegia	25(25/100)	22.97(150/653)	39.91(87/218)		19.42(47/242)	8.42(16/190)	
Quadriplegia	26(26/100)	17.46(114/653)	18.35(40/218)		13.64(33/242)	13.68(26/190)	
Mixed	3(3/100)	1.38(9/653)	16.97(37/218)		5.37(13/242)	10(19/190)	
Dyskinesia	2(2/100)	0.46(3/653)	10.55(23/218)		2.89(7/242)	7.89(15/190)	
Ataxia	4(4/100)	0.15(1/653)	0(0/218)		4.13(10/242)	0(0/190)	
Epilepsy							
☐ No	85(85/100)	87.44(571/653)	79.82(174/218)		87.19(211/242)	92.63(176/190)	0.004
☐ Yes	15(15/100)	12.56(82/653)	20.18(44/218)		12.81(31/242)	7.37(14/190)	
Hearing loss							
No	84(84/100)	95.25(622/653)	80.73(176/218)		91.32(221/242)	88.42(168/190)	< 0.001
Yes	16(16/100)	4.75(31/653)	19.27(42/218)		8.68(21/242)	11.58(22/190)	
Visual imparimen							
No	91(91/100)	88.67(579/653)	91.28(199/218)		90.5(219/242)	93.16(177/190)	0.408
Yes	9(9/100)	11.33(74/653)	8.72(19/218)		9.5(23/242)	6.84(13/190)	
Intellectual disability							
No	33.33(21/63)	66.67(292/438)	48.53(66/136)		54.25(83/153)	49.61(63/127)	< 0.001
Mild ID	50.79(32/63)	27.40(120/438)	40.44 (55/136)		35.95(55/153)	33.86(43/127)	
Severe ID	15.87(10/63)	5.94(26/438)	11.03(15/136)		9.80(15/153)	16.54(21/127)	
GMFCS							
I-II	54(54/100)	66.16(432/653)	58.26(127/218)		64.46(156/242)	64.74(123/190)	0.0049
III	22(22/100)	17.76(116/653)	7.8(17/218)		18.6(45/242)	15.79(30/190)	
IV-V	24(24/100)	16.08(105/653)	33.94(74/218)		16.94(41/242)	19.47(37/190)	
Impairment index							
Low	19.05(12/63)	46.82(206/440)	35.04(48/137)		35.29(54/153)	35.43(45/127)	0.0002
Moderate	47.62(30/63)	33.41(147/440)	34.31(47/137)		39.22(60/153)	33.07(42/127)	
High	33.33(21/63)	19.77(87/440)	30.66(42/137)		25.49(39/153)	31.5(40/127)

### MRI patterns and comorbidities

**Fig. 3:**
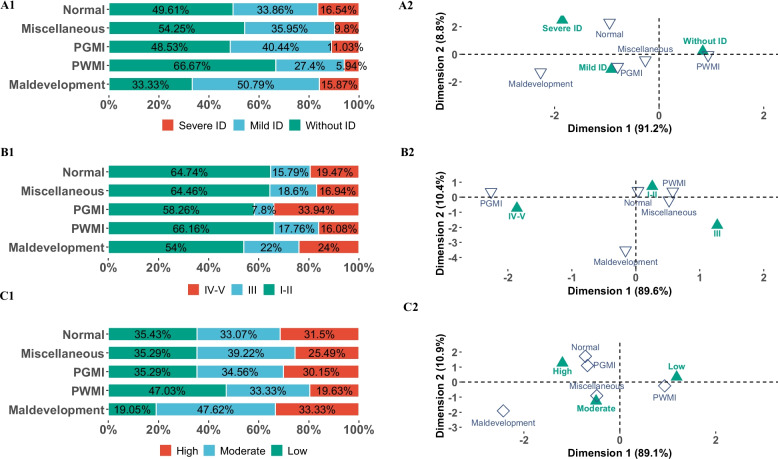
The relationship between MRICS patterns and intellectual disability, GMFCS and impairment index. **A1** The composite column chart illustrates the distribution of intellectual disability across MRICS categories. Two-thirds of children with maldevelopments had intellectual disability, followed by those with PGMI (51.5%)**.** Normal findings were associated with the highest proportion of severe intellectual disability (16.54%), followed by maldevelopments (15.87%). There were significant differences between PWMI and all other MRICS patterns in post hoc pairwise comparisons (p.adj < 0.05). **A2** The correspondence analysis figure shows the relationship between MRICS and intellectual disability. Dimension 1 appears to reflect the severity of intellectual impairment. The maldevelopment pattern was most closely associated with severe intellectual disability, whereas PWMI was most strongly associated with no intellectual disability. **B1** The composite column chart displays the distribution of GMFCS levels across MRICS groups. Overall, 63.57% (892/1403) of children were ambulatory (GMFCS levels I–II). Only the PWMI and PGMI groups differed significantly in post hoc pairwise comparisons (p.adj = 0.01). **B2** The correspondence analysis figure indicates that children with PGMI were more frequently classified into GMFCS levels IV–V, reflecting more severe motor impairment. **C1** The composite column chart presents the distribution of the impairment index across MRICS categories. Children with PWMI had the highest proportion of low impairment (47.03%), while those with maldevelopments had the lowest (19.05%). Significant differences were found between PWMI and the maldevelopment, PGMI, and normal groups in post hoc pairwise comparisons (all p.adj < 0.05). **C2** The correspondence analysis figure suggests that Dimension 1 represents overall impairment severity. The maldevelopment pattern was most associated with a high impairment index, while PWMI was most associated with a low impairment index

Comorbid conditions varied across MRI categories. Epilepsy and hearing loss were most prevalent in children with PGMI (20.2% and 19.3%, respectively), and least common in PWMI (12.6% for epilepsy, 4.8% for hearing loss) (Fig. 2B). In terms of intellectual disability, PWMI had the lowest rate (146/438, 33.33%), significantly lower than all other patterns (all p.adj < 0.05), while maldevelopments had the highest overall intellectual disability rate (42/63, 66.7%). Interestingly, severe intellectual disability was most frequent in children with normal MRI (16.54%), followed by maldevelopments (15.87%) (Fig. 3A1). Correspondence analysis showed PWMI correlated with no intellectual disability, while maldevelopments were linked with severe intellectual disability (Fig. 3 A2).

### MRI patterns and motor function

Overall, 63.6% (892/1,403) of the cohort were ambulatory (GMFCS levels I–II). Ambulation was highest among children with PWMI (66.2%), while those with PGMI showed the highest proportion of severe motor impairment (GMFCS IV–V: 33.9%). Post hoc comparisons revealed a significant difference between the PWMI and PGMI groups (p.adj = 0.01) (Fig. 3B1-B2).

### MRI patterns and impairment index

The impairment index showed clear trends across MRI patterns. Children with PWMI had the highest rate of low impairment (47%) and the lowest rate of high impairment (19.8%). Conversely, maldevelopments were associated with the highest rate of high impairment (33.3%) and the lowest rate of low impairment (19.1%). These differences were statistically significant between PWMI and all other patterns (p.adj < 0.05). Correspondence analysis supported these findings, showing that PWMI aligned with low impairment, while maldevelopments were linked to severe impairment (Fig. 3C1-C2).

## Discussion

This large hospital-based study underscores the clinical utility of MRICS in characterizing neuroimaging abnormalities in CP and linking them with functional outcomes. We found that MRI abnormalities were present in 86.5% of children with CP, with PWMI being the most common pattern. PWMI was strongly associated with favorable motor and cognitive outcomes, whereas PGMI and maldevelopments were linked with more severe clinical impairments. Notably, 13.5% of children with normal MRI findings exhibited high impairment, challenging the assumption that structurally normal imaging predicts favorable outcomes.

The high diagnostic yield of MRI in this cohort (86.5%) aligns with previous reports from population-based studies across Europe and North America, confirming its value in CP evaluation [3, 9, 20]. PWMI emerged as the most frequent MRI pattern (46.5%), similar to findings from European cohorts [10, 15], which affirms the cross-cultural applicability of the MRICS in Asian clinical settings. However, regional variability exists; a Turkish cohort, for example, reported a lower prevalence of PWMI (27.7%) [6], highlighting the influence of imaging protocols, classification methods, and referral patterns [8, 14]. WMI is the most common form of brain injury in premature infants and is a leading cause of long-term neurodevelopmental disabilities, including CP and cognitive impairments [21–23]. The pathophysiology of WMI is multifactorial and closely linked to the unique vulnerability of the immature brain [24]. During 23–32 weeks of gestation, pre-oligodendrocytes—the precursors to myelinating cells—dominate the white matter and are particularly susceptible to oxidative stress, excitotoxicity, and inflammation [25, 26]. Systemic or intrauterine inflammation can activate microglia, releasing cytokines that damage these vulnerable cells [27]. In addition, immature cerebral autoregulation in preterm infants leads to fluctuations in blood flow, predisposing periventricular regions to ischemia [28]. Mitochondrial dysfunction further exacerbates injury by impairing energy metabolism and increasing reactive oxygen species [29]. Glutamate excitotoxicity also contributes by overactivating NMDA and AMPA receptors, leading to calcium overload and cell death [30]. Even in the absence of overt necrosis, these mechanisms may disrupt oligodendrocyte maturation, leading to delayed myelination or delayed mild WMI [31].

MRI patterns demonstrated distinct clinical profiles. PWMI was most frequently associated with spastic CP subtypes—particularly diplegia and hemiplegia—and showed better gross motor outcomes. In contrast, PGMI was more commonly associated with dyskinetic and mixed CP subtypes, with higher rates of epilepsy and hearing loss. This pattern likely reflects injury to deep gray nuclei—including the basal ganglia and thalamus—which play pivotal roles in motor coordination, sensorimotor integration, and cortical regulation [32, 33]. Interestingly, PGMI was more prevalent in females—an atypical finding [34, 35], possibly reflecting survivorship bias or sex-specific genetic susceptibilities [36, 37].

The heterogeneous “miscellaneous” category warrants deeper exploration. A potential contributor within this group is cerebellar injury—an increasingly recognized pathology in premature infants that may manifest as atrophy, delayed myelination, or signal abnormalities. The cerebellum undergoes rapid growth during late gestation and is highly susceptible to hemorrhage or hypoxia in preterm neonates [38, 39]. Cerebellar damage has been linked to motor incoordination, cognitive deficits, and language delays—clinical features often seen in CP children whose imaging does not conform neatly to classical MRICS categories. Given that moderate preterm birth was significantly associated with “miscellaneous” findings (RRR = 2.20), it is plausible that subtle cerebellar injury contributes to this pattern. Future studies incorporating volumetric and advanced sequences, such as DTI and resting-state fMRI, may enhance the characterization of these cases.

The normal MRI group remains particularly noteworthy. Although structurally unremarkable, 13.5% of these children exhibited high impairment. This challenges assumptions about the prognostic value of imaging and suggests that microstructural abnormalities or genetic etiologies may be missed on conventional MRI [9, 40, 41]. Moreover, emerging evidence points to a possible genetic etiology in some of these cases, particularly when there is no history of perinatal adversity and no imaging explanation for the symptoms [42–44]. These findings underscore the need for a broader diagnostic approach—including genetic testing, metabolic screening, and neurophysiological assessment in this subgroup to uncover potential underlying mechanisms—for children with CP and normal MRI.

Comorbidities also varied by MRI pattern [45]. Children with PGMI had the highest prevalence of epilepsy and hearing loss, while those with PWMI had the lowest rates of these complications. These findings are consistent with prior work demonstrating that injury to deep gray matter structures increases the risk of seizures and sensory deficits [18, 32]. Our use of a multidimensional impairment index further revealed that PWMI was most often associated with low overall impairment, while maldevelopments had the highest proportion of children with severe impairments. This reinforces the idea that both injury type and topography influence the severity of neurodevelopmental outcomes, affecting not only motor abilities but also cognition and sensory domains.

This study has several limitations. First, MRI interpretation was based on both radiology reports and image review. In some cases, particularly among children classified with normal MRI without follow up MRI or advanced imaging, which may miss subtle lesions not emphasized in narrative interpretations. Second, the timing of imaging may have influenced classification accuracy. The median MRI age was 18 months, with nearly two-thirds of MRIs obtained before the age of 2 years—before complete myelination. As such, differentiating age-appropriate delayed myelination from pathological WMI is challenging without serial imaging or advanced modalities such as diffusion tensor imaging. Some early injuries may have undergone pseudonormalization, potentially contributing to the proportion of “normal” findings despite clinical impairment. Third, cerebellar abnormalities may have been under-detected due to limitations in conventional MRI resolution. Subtle cerebellar atrophy or developmental delay in cerebellar growth—common in preterm infants—may contribute to motor, cognitive, and language dysfunction but remain unrecognized in routine clinical MRI [38, 39]. Future studies should incorporate volumetric and functional imaging to improve detection. Finally, selection bias may exist due to exclusion of children without MRI. These exclusions, while necessary, may limit the generalizability of findings to all children with CP.

Our findings support the routine use of standardized MRI classification systems like MRICS to stratify children with CP by injury type and functional prognosis. Distinct MRI profiles reflect how lesion topography shapes neurodevelopmental outcomes. However, MRI alone is insufficient. Multimodal assessments—including advanced imaging, electrophysiology, and genetic testing—are vital, especially for children with non-specific or normal scans. Early identification of white matter or cerebellar lesions may enable timely neurorehabilitation or neuroprotection. Longitudinal studies integrating imaging, developmental follow-up, and molecular diagnostics are essential to refine CP subtypes and personalize care, particularly in cases with normal MRI where novel mechanisms may be involved.

## Conclusion

MRICS enables structured interpretation of MRI findings and correlates meaningfully with functional outcomes in children with CP. Recognizing the distinct clinical signatures of each MRI pattern can guide prognosis, optimize management, and help identify children who may benefit from additional diagnostic investigations. Future work integrating imaging with genomic and neurophysiological data may further elucidate the complex etiologies underlying CP.

## Data Availability

The data supporting the findings of this study are available from the corresponding author upon reasonable request.

## Abbreviations

CP: Cerebral Palsy
MRI: Magnetic Resonance Imaging
MRICS: MRI Classification System
PWMI: Predominant White Matter Injury
PGMI: Predominant Gray Matter Injury
GMFCS: Gross Motor Function Classification System
RRR: Relative Risk Ratio
SE: Standard Error
CI: Confidence Interval
